# Diversity of Intestinal Bacterial Microbiota of Indigenous and Commercial Strains of Chickens Using 16S rDNA-Based Analysis

**DOI:** 10.3390/ani10030391

**Published:** 2020-02-28

**Authors:** Waleed Al-Marzooqi, Zeyana A.S. Al-Maskari, Kaadhia Al-Kharousi, Eugene H. Johnson, Yasmin El Tahir

**Affiliations:** Department of Animal and Veterinary Sciences, College of Agricultural and Marine Sciences, Sultan Qaboos University, P.O. Box 34, Al-Khoud 123, Omankaadhia@squ.edu.om (K.A.-K.); ejohnson@squ.edu.om (E.H.J.); yasmin@squ.edu.om (Y.E.T.)

**Keywords:** intestinal, microflora, 16S rDNA, strain, chickens

## Abstract

**Simple Summary:**

Little is known about how the bacterial community differs among different genetic breeds of chickens, especially those with various growth rates, such as in local native chickens. Our data, generated by molecular detection revealed the heterogeneity of bacterial populations existing in different intestinal segments for the two strain of chickens.

**Abstract:**

The objective of this study was to assess the relative abundance of bacteria microflora in different segments of the gastrointestinal tract (duodenum, jejunum, ilium, and cecum) of indigenous (local Omani) and commercial (Cobb 500) chicken strains. Birds were raised under an intensive management system fed a nonmedicated corn-soybean meal diet from Day 0–35 days of age. Using 16S rDNA-based analysis the study showed that in both breeds of birds Bacilli were the most abundant class of bacteria in the duodenum, jejunum, and ileum. Local Omani chickens had significantly higher numbers of Clostridia at most time periods. Actinobacteria were found in higher numbers and reached 54.9% of the bacteria in the jejunum at Day 35 in Cobb 500 versus only 5.42% in the Omani chickens. The bacterial microbiota relative abundance differed significantly (*p* < 0.05) across different intestinal segments of the two strains, suggesting that each region developed its own bacterial community and the relative abundances of these were quite different.

## 1. Introduction

The study of gut microbiota in birds is finally beginning to blossom within the present scientific literature. A number of studies have revealed not only their importance in nutritional utilization, immunological priming of the intestinal tract but also their role as a driving force in growth and development. 

The composition of the intestinal microflora in chickens continues to change over time under the influence of different factors, such as bird age and dietary factors [1,2]. There is also significant diversity in bacterial populations among different parts of the digestive tract of the birds [3]. Each region of the GI tract develops its own unique microbial profile, and the composition of the microflora becomes more complex and changes in relation to the age of the chickens, different feed ingredients [4,5,6], breed and geographic location [7,8,9]. 

With the development of animal husbandry in Oman, local “indigenous” chicken production is becoming more widespread. Local chicken production is among the farming activities in the rural communities of Oman that provides opportunities for food security and income for many rural families [10]. An improvement in local chicken productivity would be highly valuable in the enhancement of the socioeconomic and nutritional status of farmers. Numerous studies have shown the beneficial effect of the microbial community in the gastrointestinal tract of the host and their important contributions in many roles such as in nutrient absorption, feed digestion, and immune system [4]. 

The development of the intestinal bacteria of different genetic lines of chickens has become a recent point of interest [11]. However, little is known about how the bacterial community varies among different genetic strains of chickens, especially those with various growth rates such as local Omani chickens. 

The use of modern approaches, that involve analyzing the structure of bacterial communities by determining the characteristic features of the microbial DNA extracted from the community samples, has overcome the difficulties in culturing of individual microbes [12,13,14,15]. Using such techniques, it has been found that 90% of the bacteria in the chicken gastrointestinal tract represent previously unknown species [13]. In addition, metagenomics; a nonculture-based approach, was developed and enabled researchers to comprehensively study microbial communities in different ecosystems [16]. Metagenomic analysis has provided significant information on the changes/succession in the microbial community [16].

Understanding of the development, diversity, and succession of bacterial community will permit detecting disruption in the microbiota. This information may enable manipulating the intestinal flora to enhance the intestinal health and bird performance in general. The objective of this study was to assess the relative abundance of bacteria microbiota identified in the intestinal tract of local Omani and Cobb 500 broiler chickens fed a nonmedicated conventional corn-soybean meal diet from 0 to 35 days of age using 16S rDNA-based analysis.

## 2. Materials and Methods

### 2.1. Ethical Approval

The study was approved by the Animal Research Ethics Board at Sultan Qaboos University (Ethical Code: IG/AGR/ANVS/15/02). All experimental work was conducted at the Poultry Research Unit at the Agricultural Experiment Station in accordance with the experimental unit policy on animal welfare and the requirements of the procedures involving animals/birds and their care were conducted in conformity with international laws and policies (EEC Council directives 86/609, OJL 358, 1 December, 12, 1987; NIH Guide for the Care and Use of Laboratory Animals, NIH Publications No. 85-23, 1985) at Sultan Qaboos University.

### 2.2. Bird and Housing

One hundred and fifty 1-day-old chicks from each strain of indigenous chickens (local Omani) and Cobb 500 broiler strain were obtained from reputable commercial hatcheries at Barka in Muscat. Chicks were inspected on arrival to ensure that all chicks were free from deformities and early sign of disease. Standard Operating Procedures of broiler house management [17] was followed throughout the experiment. Cleaning and disinfecting closed house unit, cages, feeders, and drinkers through fumigation were performed before the experiment. In addition, strict hygiene and biosecurity measurements were implemented. On the day of arrival, the chicks were individually weighed and placed into narrow weight classes. Birds of relatively low or high body weight were excluded. Six birds were randomly assigned to each of 30 suspended wire cages (62 × 62 × 37 cm) such that all cages had nearly a similar average initial weight. Feed was available ad libitum. The cages were in an environmentally controlled shed (closed house) maintained at 33 °C on Day 1 and reduced by 3 °C each week to reach a constant 22 °C. The lighting was 23L: 1D. 

### 2.3. Experimental Diets

Chicks of both strains were fed a nonmedicated conventional corn-soybean meal diet; i.e., devoid of other dietary supplements that may influence the microflora’s composition, from Day 0–35 days of age. The composition of experimental diet is as described by Al-Marzooqi et al. [10]. There were 15 replicates for each strain of chicken with each replicate cage containing six birds (a total of 90 birds/strain). Birds per replicate combinations were randomly allocated.

### 2.4. Collection of Gastrointestinal Tract Contents

At 5, 15, 25, and 35 days of age, one bird per cage for each strain of chicken was randomly selected. Selection was based on the body weight of the birds and birds with the body weight closest to the average from each cage were selected, marked, and kept in their cage until being euthanized. Then, the selected bird was injected with a mix dose of ketamine (Ketamil Injection—Ketamine 100 mg/mL (as hydrochloride) Troy Laboratories Pty Limited, Glendenning, Australia) 10% and xylazine (Ilium Xylazil-20—Xylazine 20 mg/mL (as hydrochloride) Troy Laboratories Pty Limited) 20% intramuscularly to put the bird into a deep plan of sedation and anaesthesia. When the bird was completely immobilized, it was euthanized by cervical dislocation. Then, an incision was made at the bottom of the breastbone and a large V shape was cut towards the head. At the V shape the abdomen cavity was opened up taking care not to rupture the intestine below. Once a large enough opening had been made up, the small intestine was carefully pulled out from the abdominal cavity until the ileal-caecal-colonic junction could be seen. The duodenum (from gizzard to entry of the bile and pancreatic ducts), jejunum (from entry of the ducts to yolk stalk), ileum (from yolk stalk to ileocecal junction), and caecum (two horns) were differentiated, separated by serial bowel clamps and were cleaned using 70% alcohol wipes. Sections approximately 4 cm long (including digesta) were cut from the mid regions of the duodenum, jejunum, ileum, and ceca. Dissecting instruments were cleaned with 70% ethanol after use on each bird. The entire process of collecting intestinal contents was performed on a thoroughly cleaned workbench and required less than 30 min.

The contents collected from four parts of intestine into a labeled sterile 15-mL tube. Samples were placed on ice and immediately transported to the laboratory and stored in −80 °C freezer until analysis. Analysis of all samples were started two weeks after the end of the experiment at Day 35. The conditions were the same for all the collected samples.

### 2.5. DNA Extraction

Total DNA was extracted from contents of each luminal content samples (duodenum, jejunum, ileum, and cecum) using a QIAamp DNA Stool Mini Kit (QIAGEN, CA, Hamburg, Germany) according to the manufacturer’s instructions. The DNA concentration was evaluated by measuring optical density using Nano-Drop 2000 (Thermo Electron Corporation, Waltham, MA, USA) at a wavelength of 260 and 280 nm. The integrity of the DNA extracts was assessed visually using 1.0% agarose gel (containing ethidium bromide) electerophoresis. 

### 2.6. Polymerase Chain Reaction Amplicon Production and High-Throughput Sequencing

The variable regions V3-V4 of the 16S rDNA gene were amplified and sequenced. The PCRs were performed in triplicate in a total volume of 20 μL containing 5 μM of each primer, 10 ng of DNA template, 4 μL 1× FastPfu buffer, 2.5 mM dNTPs, and 0.4 μL of FastPfu polymerase (TransGen Biotech, Beijing, China). PCR conditions were as follows: Initial denaturation at 95 °C for 2 min; followed by 25 cycles of denaturation 94 °C for 30 s, annealing at 55 °C for 30 s, and extension at 72 °C for 30 s and then, a final extension at 72 °C for 5 min. PCR products were separated on 2% agarose gels, and purified using the DNA gel extraction kit (Axygen Scientific Inc., Union City, CA, USA). Amplicons produced form different intestinal luminal content samples were sent to a commercial company (BGI Genomic Lab, Tai Po Industrial Zone, New Territories, Hong Kong, China) for sequencing on the Illumina MiSequencing platform.

### 2.7. Sequencing Analysis

All the raw sequences obtained from Illumina Miseq were firstly filtered for quality control to get operational sequences. The quality control and analysis of the sequences were performed using the software Quantitative Insights into Microbial Ecology (QIIME, v1.8.0) [18]. The paired-end reads from the DNA fragments were merged using FLASH [19]. Sequences data was treated by read trimming and identification of V3-V4 sequences and set of sequences with ≥97% identity were defined as an operational taxonomic unit (OTU). The UCLUST [20] clustering method was used to cluster operational taxonomic units. The defined OTUs were assigned to different taxonomic levels (phylum, class, genus and families) at a cutoff of 97%. The clustered OTUs were also used to construct the rarefaction curves and calculate the Shannon and Simpson diversity indices, abundance-based coverage estimators (ACE), Chao 1 richness, and coverage percentage by Good’s method. 

### 2.8. Bioinformatics and Statistical Analyses

Bioinformatics and statistical analyses were performed using the QIIME and R package (v3.1.1). The alpha-diversity indices (Chaol, ACE, Shannon diversity index, and Simpson index) were calculated to establish the relative abundance and diversity of the sequences. Beta diversity was determined using unweighted Unifrac distance metrics to evaluate the structure and distribution of the microbial genetic communities among the samples. Differences in the Unifrac distances for pairwise comparisons among groups were calculated using Student’s t-test and the Monte Carlo permutation test with 1000 permutations. Metastats and R package (v3.1.1) [21] were used to compare and determine which taxonomic groups were significantly different between groups of samples based on intestinal segments and age period. The differences were considered to be significant at *p* < 0.05. The obtained *p*-value was adjusted by a Benjamini–Hochberg false discovery rate correction (Function ‘p.adjust’ in the stats package of R (v3.1.1)). 

## 3. Results

Our data obtained for both breeds of chicken showed that each region of intestinal segments developed its own bacterial community and the diversity of the bacterial community changed from one age period to the next. In addition, Bacilli were the dominant 16S rDNA sequences in the duodenum, jejunum, and ileum libraries, whereas Clostridia were the dominant 16S rDNA sequences in the cecum libraries. 

The most intriguing observation in this study is that at the early stage of their lives (five days of age), local Omani chickens had a significantly a smaller percentage of Bacilli in their duodenum (68.5%) when compared to Cobb 500 broiler chickens (98%), whereas Clostridia at the same age was more dominant in the Omani local chickens (25.84%) compared to Cobb 500 broiler chickens (0.88%). The microbial composition of each intestinal segment at different ages for each strain of chicken was summarized in their respective section below.

### 3.1. Microbial Composition of the Duodenum of Cobb 500 Broiler Chickens

Bacteria classified according to their respective Phylum and Class, found in the duodenum of broiler chickens at different ages, are presented in Table 1. Thirty bacterial microbiota at the Class level were found in the duodenum. Of the 275,030 reads, Bacilli were most abundant, at 88% of the total sequences. Actinobacteria and Proteobacteria represented a small percentage of 4.36% and 4.08%, respectively, of the total sequences. Clostridia accounted for 2.87% of the total sequences. Across different age periods, Bacilli were the dominant group, representing 98.01% at Day 5, 94.93% at Day 15, 73.34% at Day 25 to 85.15% at Day 35 of the sequences. Clostridia sequences fluctuated from 0.88% at Day 5, 1.68% at Day 15, 8.34% at Day 25, and 0.85% at Day 35. Actinobacteria sequences were Day 5: 0.18%, Day 15: 0.58%, Day 25: 16.77%, and Day 35: 0.49%, while sequences related to Erysipelotrichia were Day 5: 0.03%, Day 15: 0.01%, Day 25: 0.58%, and Day 35: 0.0%. Proteobacteria group-related sequences were detected at smaller percentages across all age periods except for Gammaproteobacterial detected at 10.29% at Day 35 of age. 

### 3.2. Microbial Composition of the Jejunum of Cobb 500 Broiler Chickens

Bacteria classified according to their respective Phylum and Class, found in the jejunum of broiler chickens at different ages are presented in Table 2. Thirty bacterial microbiota at the Class level were found in the jejunum. Of the 212,094 reads, Bacilli were the most the abundant, at 76.79% of the total sequences. Clostridia and Proteobacteria represented a small percentage of 2.62% and 2.45%, respectively of the total sequences. Actinobacteria accounted for 17.53% of the total sequences. Across different age periods, Bacilli were the dominant group, representing 88.83% at Day 5, 93.09% at Day 15, 93.03% at Day 25, and 39.05% at Day 35 of the sequences. Clostridia sequences fluctuated from 5.72% at Day 5, 1.45% at Day 15, 0.40% at Day 25, and 3.35% at Day 35. Actinobacteria sequences were Day 5: 1.52%, Day 15: 0.92%, Day 25: 5.31%, and Day 35: 54.89%, while sequences related to Erysipelotrichia were Day 5: 0.16%, Day 15: 0.02%, Day 25: 0.02%, and Day 35: 0.01%. Proteobacteria group-related sequences were detected at smaller percentages across all age periods. 

### 3.3. Microbial Composition of the Ileum of Cobb 500 Broiler Chickens

Bacteria classified according to their respective Phylum and Class, found in the ileum of broiler chickens at different ages are presented in Table 3. Thirty bacterial microbiota at the Class level were found in the ileum. Of the 200,624 reads, Bacilli were the most abundant, at 75.74% of the total sequences. Actinobacteria and Proteobacteria sequences represented 5.89% and 6.38%, respectively of the total sequences. Clostridia accounted for 11.73% of the total sequences. Across different age periods Bacilli were the dominant group, representing 98.33% at Day 5, 88.13% at Day 15, 42.97% at Day 25 to 67.81% at Day 35 of sequences. Clostridia sequences fluctuated from 0.81% at Day 5, 5.93% at Day 15, 24.49% at Day 25, and 18.62% at Day 35. Actinobacteria sequences were Day 5: 0.51%, Day 15: 1.54%, Day 25: 11.62%, and Day 35: 11.41%, while sequences related to Erysipelotrichia were Day 5: 0.01%, Day 15: 0.07%, Day 25: 0.17%, and Day 35: 0.02%. Proteobacteria group-related sequences were detected at smaller percentages across all age periods. 

### 3.4. Microbial Composition of the Cecum of Cobb 500 Broiler Chickens

Bacteria classified according to their respective Phylum and Class, found in the cecum of broiler chickens at different ages are presented in Table 4. Thirty bacterial microbiota at the Class level were in the cecum. Of the 208,941 reads, Clostridia were the most abundant, at 89.42% of the total sequences. Only a few of Coriobacteria (1.09%) and Erysipelotrichia (2.52%) related to the total sequences were detected. Actinobacteria and Proteobacteria represented a very small percentage of 0.20% and 0.020%, respectively, of the total sequences. Bacilli accounted for 6.59% of the total sequences. Across different age periods Clostridia were the dominant group, representing 80.61% at Day 5, 92.19% at Day 15, 91.26% at Day 25 to 94.19% at Day 35 of the sequences. Bacilli sequences fluctuated from 15.93% at Day 5, 5.64% at Day 15, 2.97% at Day 25, and 0.56% at Day 35. Erysipelotrichia sequences were Day 5: 2.84%, Day 15: 0.47%, Day 25: 5.09%, and Day 35: 2.33%. Actinobacteria sequences were Day 5: 0.0%, Day 15: 0.0%, Day 25: 0.01%, and Day 35: 0.82%. Proteobacteria group-related sequences were detected at smaller percentages across age periods. 

### 3.5. Differences of Microbial Communities among Samples from Different Intestinal Segments of Cobb 500 Broiler Chickens

The *p*-value distribution of 16S rDNA gene sequence libraries comparing the quantitative differences of microbial communities among samples from different intestinal segments of broiler chickens are presented in Table 5. Statistical comparisons of the libraries revealed that the composition of the Duodenum-Jejunum, Duodenum-Ileum, Cecum-Duodenum, Cecum-Ileum Cecum-Jejunum bacterial microbiota differed significantly (*p* < 0.05), suggesting that each region developed its own bacterial community. The number of Actinobacteria, Coriobacteria, Bacilli, Clostrdia, Erysipelotrichia, Alphaproteobacteria, Betaproteobacteria, Deltaproteobacteria, and Gammaproteobacteria differed significantly across different intestinal segments (*p* < 0.05). Bacilli were the dominant 16S rDNA sequences in the duodenum, jejunum, and ileum libraries, whereas Clostridia were the dominant 16S rDNA sequences in the cecum libraries. 

### 3.6. Differences of Microbial Communities among Samples from Cobb 500 Broiler Chickens of Different Age Groups

The *p*-value distribution of 16S rDNA gene sequence libraries comparing the quantitative differences of microbial communities among samples from broiler chickens at different age groups are presented in Table 6. Statistical comparisons of the libraries revealed that there were no significant differences (*p* > 0.05) between the microbial composition at different age groups: Day 5–15, Day 5–25, Day 5–35, Day 15–25, Day 15–35, and Day 25–35. The results of the statistical evaluation at certain age groups revealed that the percentage of bacterial microbiota of Acidobacteriia, Betaproteobacteria, and Cytophagia varied significantly (*p* < 0.05). The average percentage of Acidobacteriia detected at Day 25 (0.01%) of age was significantly higher (*p* < 0.05) than at Day 35 (0.001%) of age. The average percentage of Betaproteobacteria was detected at significantly higher levels (*p* < 0.05) at Day 35 (0.67%) of age than at Day 5 (0.05%) of age. The average percentage of Cytophagia at Day 15 (0.02%) was significantly different (*p* < 0.05) from those of the other age groups.

### 3.7. The Taxonomic Composition Distribution of the Bacterial Community in Intestinal Segments of Cobb 500 Broiler Chickens

**Figure 1:**
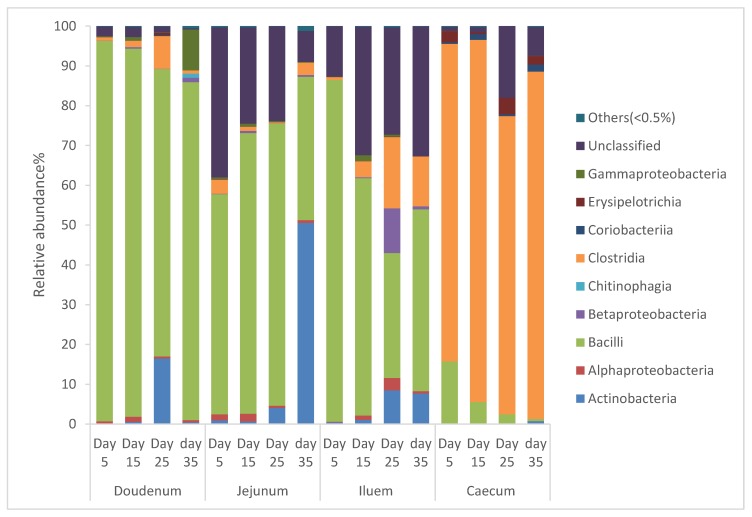
Percentage of relative abundance of bacterial community of Cobb 500 broiler chickens determined from different intestinal segments at different age periods from 16S rDNA libraries.

From Figure 1, it can be seen that the diversity of the bacterial community in the intestinal segments of broiler chickens changed from one age period to the next. Species that exhibited an abundance less than 0.5% in all samples were classified into “others”. The intestinal segments of duodenum, jejunum, and ileum had a higher relative abundance of Bacilli, and as the birds aged, the percentage of Bacilli decreased, whereas the cecum had a higher relative abundance of Clostridia and as the birds aged, the percentage of Clostridia increased. 

### 3.8. Microbial Composition of the Duodenum of Local Omani Chickens

Bacteria classified according to their respective Phylum and Class, found in the duodenum of local Omani chickens at different ages are presented in Table 7. Thirty-five bacterial microbiota at the Class level were found in duodenum. Of the 391,488 reads, Bacilli were the most abundant, at 76.8% of the total sequences, while sequences related to Clostridia accounted for 15.4% of the total sequences. Proteobacteria accounted for 5.93% of the total sequences. Actinobacteria, Coriobacteria, Rubrobacteria, Thermoleophilia, Flavobacteria and Deinococci and Tissierellia represented a very small percentage of 0.97%, 0.01%, 0.004%, 0.003%, 0.06%, 0.14%, 0.04%, and 0.03%, respectively of the total sequences. Across different age periods Bacilli were the dominant group, representing 68.52% at Day 5, 86.91% at Day 15, 61.19% at Day 25 to 92.83% at Day 35 of the sequences. Clostridia sequences fluctuated from 25.84% at Day 5, 6.64% at Day 15, 26.75% at Day 25, and 0.89% at Day 35. Both Actinobacteria and Erysipelotrichia related sequences were Day 5: 0.4%, Day 15: 0.55%, Day 25: 0.50% and Day 35: 2.42% and Day 5: 0.56%, Day 15: 0.09%, Day 25: 0.15%, and Day 35: 0.02%, respectively. Proteobacteria group-related sequences were detected at lower levels across age periods except for Alphaproteobacteria and Gammaproteobacteria detected at Day 5Day 5: 1.65%, Day 15: 1.82%, Day 25: 1.42% and Day 35: 2.03% and (Day 5Day 5: 1.71%, Day 15: 2.14%, Day 25: 7.44%, and Day 35: 0.0.56%, respectively). 

### 3.9. Microbial Composition of the Jejunum of Local Omani Chickens

Bacteria classified according to their respective Phylum and Class, found in the jejunum of local Omani chickens at different ages are presented in Table 8. Thirty-five bacterial microbiota at the Class level were found in the jejunum. Of the 307,792 reads, Bacilli were the most abundant, at 89.91% of the total sequences. Clostridia and Actinobacteria represented 6% and 3.09% of the total sequences. Proteobacteria accounted for 0.65% of the total 16S rDNA sequences. Coriobacteria, Rubrobacteria, Thermoleophilia, Chitinophagia, Flavobacteria, Deinococci, and Tissierellia represented a very small percentage at 0.02%, 0.01%, 0.01%, 0.001%, 0.001%, 0.01%, 0.004%, respectively of the total sequences. Across different age periods Bacilli were the dominant group, representing 89.06% at Day 5Day 5, 89.16% at Day 15, 94.92% at Day 25 to 86.09% at Day 35 of the sequences. Clostridia sequences fluctuated from 4.49% at Day 5, 8.61% at Day 15, 3.65% at Day 25, and 7.71% at Day 35. Actinobacteria sequences were Day 5: 5.25%, Day 15Day 15: 0.61%, Day 25: 0.89%, and Day 35: 5.42%, while Erysipelotrichia sequences were Day 5: 0.11%, Day 15Day 15: 0.15%, Day 25: 0.04%, and Day 35: 0.06%. Proteobacteria group-related sequences were detected at smaller amounts across all age periods. 

### 3.10. Microbial Composition of the Ileum of Local Omani Chickens

Bacteria classified according to their respective Phylum and Class, found in the ileum of local Omani chickens at different ages are presented in Table 9. Thirty-five bacterial microbiota at the Class level were found in the ileum. Of the 268,837 reads, Bacilli were the most abundant, at 82.10% of the total sequences. Actinobacteria and Proteobacteria sequences were less abundant in the ileum and detected at 1.45% and 1.32%, respectively of the total sequences. Clostridia accounted for 14.81% of the total sequences. Across different age periods Bacilli were the dominant group, representing 86.44% at Day 5, 85.19% at Day 15Day 15, 71.15% at Day 25 to 85.68% at Day 35 of the sequences. Clostridia sequences fluctuated from 9.23% at Day 5, 13.01% at Day 15Day 15, 26.10% at Day 25, and 10.29% at Day 35. Actinobacteria sequences were Day 5: 1.39%, Day 15Day 15: 0.83%, Day 25: 0.76%, and Day 35: 3.48%, while Erysipelotrichia sequences were Day 5: 0.21%, Day 15Day 15: 0.07%, Day 25: 0.14%, and Day 35: 0.05%. Proteobacteria group-related sequences were detected at smaller amounts across all age periods. 

### 3.11. Microbial Composition of the Cecum of Local Omani Chickens

Bacteria classified according to their respective Phylum and Class, found in the duodenum of local Omani chickens at different ages are presented in Table 10. Thirty-five bacterial microbiota at the Class level were found in the cecum. Of the 266,937 reads, Clostridia were the most abundant, at 81.13% of the total sequences. Erysipelotrichia and Proteobacteria represented a small percentage of 2.09% and 2.94%, respectively of the total sequences. Only a few Actinobacteria (0.73%) related sequences were detected. Bacilli accounted for 4.87% of the total sequences. Across different age periods Clostridia were the dominant group, representing 79.08% at Day 5, 93.48% at Day 15Day 15, 85.55% at Day 25 to 69.14% at Day 35 of the sequences. Bacilli sequences fluctuated from 15.45% at Day 5, 0.53% at Day 15, 0.66% at Day 25, and 0.74% at Day 35. Erysipelotrichia sequences were Day 5: 2.01%, Day 15Day 15: 3.00%, Day 25: 2.42%, and Day 35: 1.11%, while Actinobacteria sequences were Day 5: 0.02%, Day 15Day 15: 0.13%, Day 25: 2.75%, and Day 35: 0.20%. Proteobacteria group-related sequences were detected at smaller percentages across all age periods. 

### 3.12. Differences Of Microbial Communities among Samples from Different Intestinal Segments of Local Omani Chicken

The *p*-value distribution of 16S rDNA gene sequence libraries comparing the relative abundance differences of microbial communities among samples from different intestinal segments of local Omani chickens are presented in Table 11. Statistical comparisons of the libraries revealed that the composition of the Duodenum-Jejunum, Duodenum-Ileum, Cecum-Duodenum, Cecum-Ileum Cecum-Jejunum bacterial microbiota differed significantly (*p* < 0.05), suggesting that each region developed it is own bacterial community. The relative abundance of Alphaproteobacteria, Bacilli, Betaproteobacteria, Chitinophagia, Closterdia, Coriobacteriia, Cytophagia, Deinococci, Deltaproteobacteria, Eryispelotrichia, Flavobacteria, Gammaproteobacteria, Rubrobacteria, Thermoleophilia, and Tissierellia differed significantly across different intestinal segments (*p* < 0.05). Bacilli were the dominant 16S rDNA sequences in the duodenum, jejunum, and ileum libraries, whereas Clostridia were the dominant 16S rDNA sequences in the cecum libraries. 

### 3.13. Differences of Microbial Communities among Samples from Local Omani Chickens of Different Age Groups

The *p*-value distribution of 16S rDNA gene sequence libraries comparing the quantitative differences of microbial communities among samples from local Omani chickens of different age groups are presented in Table 12. Statistical comparisons of the libraries revealed that there were no significant differences (*p* > 0.05) between the microbial composition at different age groups: Day 5–15, Day 5–25, Day 5–35, Day 15Day 15–25, Day 15Day 15–35, and Day 25–35. The results of the statistical evaluation at certain age groups revealed that the percentage of the bacterial microbiota of Acidimicrobiia and Fusobacteriia varied significantly (*p* < 0.05). At Day 5 of age the percentage of Acidimicrobiia (0.002%) was significantly higher (*p* < 0.05) than at Day 15Day 15 (0.0%) and Day 35 (0.0%) of age. Fusobacteriia was detected at significantly higher levels (*p* < 0.05) at Day 35 of age (0.011%) than at Day 5 (0.0%) of age.

### 3.14. The Taxonomic Composition Distribution of the Bacterial Community in Intestinal Segments at the Class-Level of Local Omani Chickens

**Figure 2:**
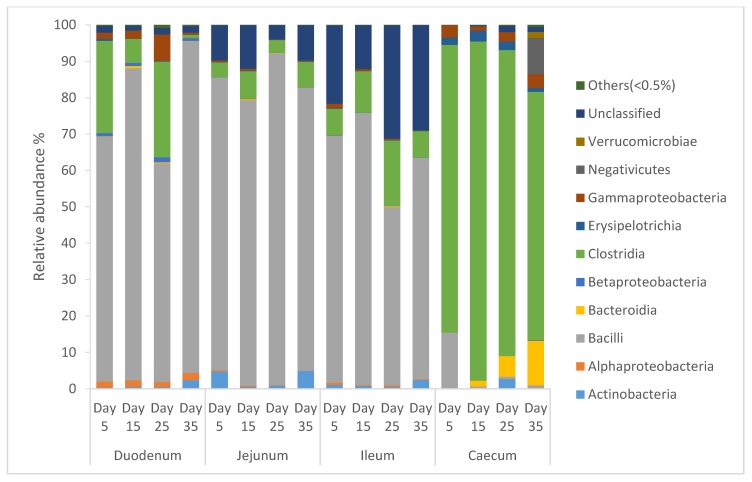
Percentage of relative abundance of bacterial community of local Omani chickens determined from different intestinal segments at different age periods from 16S rDNA libraries.

From Figure 2, it can be seen that the diversity of the bacterial community of intestinal segments of local Omani chickens changed from one age period to the next. Species of that exhibited an abundance less than 0.5% in all samples were classified into “others”. The intestinal segment of duodenum, jejunum, and ileum had a higher abundance of Bacilli, and as the birds aged, the percentage of Bacilli decreased, whereas the cecum had a higher abundance of Clostridia, and as the birds aged, the percentage of Clostridia increased. 

## 4. Discussion

Our data generated by molecular detection and bioinformatics analysis revealed the heterogeneity of bacterial populations existing in different intestinal segments. The 16S rDNA analysis generated a huge database that is beyond the scope of this manuscript. Hence, the findings of the present study have been limited to those classes of bacteria of the most quantitative significance. 

In the present study, the statistical comparisons of the libraries of microbial communities within each respective breed among samples from different intestinal segments at different age groups revealed that there were no significant differences (*p* > 0.05). On the contrary, bacterial microbiota differed significantly (*p* < 0.05) within each respective breed across different intestinal segments, suggesting that each region developed its own bacterial community and the relative abundances of these were quite different. Similar findings were represented by different studies that showed that the bacterial community is relatively transient and is replaced by a stable bacterial community once the rate of the intestinal development decreased [6,9,11].

In the duodenum of both the Cobb 500, as well as in the Omani chickens, Bacilli were the predominate class of bacteria at all age periods. However, an important difference between the two birds was the fact that local Omani chickens had significantly higher numbers of Clostridia. Indeed, at Day 5 and Day 25 Clostridia comprised 25.8% and 26.75% of the bacteria in the duodenum of local Omani chickens, respectively, versus only 0.88% and 8.388% in the Cobb 500.

It can be clearly hypothesized that the superior performance of the Cobb 500 is associated with the higher proportion of Bacilli in the duodenum or conversely that Clostridia might limit optimal duodenum development in Omani chickens.

A similar microbial picture was mirrored in the duodenum with Bacilli being the predominate class of bacteria in both birds but again with Clostridia being more abundant at Day 15Day 15, 25, and 35 in the Omani chickens. At Day 15Day 15 and 25 the Omani chickens had a 6- and 9-fold greater number of Clostridia, respectively.

In the ileum it was again observed against a backdrop of an overall higher number of Bacilli of both birds Clostridia at Day 5 made up 9.23% of the bacteria versus 0.805% in Cobb 500. This trend continued at Day 15Day 15 with Clostridia comprising 13% of the bacteria of the Omani chickens versus 5.9% in the Cobb 500. 

An interesting observation in both the duodenum and jejunum was the high number of Actinobacteria in the Cobb 500 and in the jejunum at Day 25 and 35. Indeed, at Day 25 the Actinobacteria made up 54.89% of the bacteria. This very high number of Actinobacteria is compelling and in harmony with reports of Actinobacteria being important in the upper digestive tract of other high producing broilers, such Ross 308 [22]. Again, this observation would suggest that smaller numbers of Actinobacteria in Omani chickens is not in keeping with optimal development of these intestinal segments. 

Since the function of each intestinal segment differ and provide different environmental conditions, it is expected that distinct microbiota develop in different intestinal segments [23,24]. In the present study the most profound differences in the microbial population in the intestinal segments of the two breeds was observed between Day 5 to 25. One possible explanation is that at early age periods, immediately after hatching, is the most critical period in the life of the chick. During this period, there is the transition from yolk to oral nutrition associated with major physical and functional development of the digestive tract and organs [25] resulting in unstable environmental conditions of the digestive tract’s microecosystem. However, by three weeks of age, the rate of digestive tract development lessens, and the intestinal bacterial community becomes more stable and establishes optimal environmental conditions, allowing for the clear separation of bacterial communities [4]. 

The observations of the present study permit the suggestion that Bacilli contribute more favorably to intestinal villi development and hence better nutrient absorption during the critical early period in the life of chickens. Some studies have already shown that the ileum was the most reactive site in which villus area changes significantly with nutritional changes or with an increase in nutrient content in the intestinal lumen [26,27]. 

Furthermore, Clostridia are primarily involved in fermentation [28]. The significantly larger number of Clostridia in the duodenum of local Omani chickens during early intestinal development likely have no or limited function in nutrient turn over and absorption and subsequently the duodenum functions at an impaired level when compared to that of Cobb 500 chickens.

It is noteworthy that the chicken cecum and its mucosal tissue are dominated by Clostridia related species [29,30,31]. It can be hypothesized that the higher abundance of Clostridia that are associated with fermentation act in an inhibitory fashion for nutrient absorption in the intestine of the local Omani chickens and the villus development is slowed down. The morphological analysis in a study by Al-Marzooqi et al. [10] revealed that Cobb 500 broilers had a greater villi height compared to the Omani breed (*p* < 0.01). It is assumed that an increased villus height is paralleled by an increased digestive and absorptive function of the intestine due to increased absorptive surface area, expression of brush border enzymes, and nutrient transport systems [32]. Enterocyte enzymatic activity and structure are two of the most important features of the intestinal mucosal physiology [33]. Al-Marzooqi et al. [10] concluded that villus development has a profound effect on the growth performance of the chickens. A study by Lumkins et al. [11] showed that differences in bacterial communities between the modern and ACR broiler lines may be due to variances in villi height, which could lead to an increase in the distance of the crypt away from the lumen with greater villus height, providing a niche for certain bacteria [34,35]. Thus, villi height can be considered another factor contributing to the bacterial community’s niche. 

Therefore, optimal growth and development of the intestinal tract of chicken is likely governed by early development of the intestinal tract. From this data, it would be assumed that optimal development of small intestinal microflora is associated with a preponderance of Bacilli (98%) in the duodenum at Day 5 of Cobb 500 chickens and that the inferior growth rate of the local Omani chickens was assumed to be related to the smaller number of Bacilli (68.5%) in their duodenum at Day 5. Similarly, a small number of Clostridia (0.88%) in the duodenum of the Cobb 500 and the relatively larger number (25.8%) in the duodenum at Day 5 in the local Omani chickens are reflective of the superior and inferior performance of the breeds, respectively.

Fast growth rates and improved weight gains and feed efficiency as a result of genetic selection has helped the broiler industry by providing birds that grow to a heavier weight in a shorter period of time, which have also resulted in changes to the micro-ecosystem in the gastrointestinal tract that provides stable environmental conditions to indigenous intestinal bacteria [36].

Our knowledge of gut microbiota remains incomplete. However, it should be noted that a variety of molecular techniques; such as metagenomic, next generation sequencing (NGS) or marker- gene and metagenomic sequencing (MGS), are available to investigate chicken gut microbial community composition and functions, each with different strengths and weaknesses [37]. Direct comparison among studies is difficult and may not be accurate because of variation in platforms used, breed of chickens, sampling method, differences in approaches, and concepts of studies. The high cost of sequencing analysis and the bias of some of the protocols towards detecting some taxa over others are drawbacks of these approaches. However, multiple PCR combined with q-PCR might be the tool to overcome these constrains [38]. 

## 5. Conclusions

The present study is the first to compare the microbial diversity in the small intestine and cecum of indigenous and commercial strain of chickens. Our study indicate that each region of different intestinal segments developed its own bacterial community and the relative abundances of these were quite diverse. The local breed of chicken had a 6- to 9-fold greater number of Clostridia in comparison to the commercial one and therefore, it was concluded that Clostridia were not conducive to optimal development of the intestinal villi and consequently nutrient turnover and absorption, resulting in inferior performance of the local breed. However, understanding the dynamics of the gut microbial community or microbial balance is far from complete. Future work should be directed towards identifying gut bacteria that can be associated with improved/poor chicken performance. In addition, future studies need to be directed towards development of diets such as the utilization of probiotics or manipulation of fiber intake to increase the development of Bacilli during early intestinal development and subsequent better utilization of nutrients in the local chickens.

## Figures and Tables

**Table 1:** Abundance of bacterial 16S rDNA sequences (*n* = 275,030) identified from the duodenum microbiota of Cobb 500 broiler chickens.

Phylum	Class	Abundance of Sequence (No. of Sequence [%]) Day:			
		Day 5	Day 15	Day 25	Day 35
Acidobacteria	Acidobacteriia	1 (0.001)	0 (0)	3 (0.0045)	0 (0)
	Blastocatellia	4 (0.006)	0 (0)	0 (0)	0 (0)
Actinobacteria	Acidimicrobiia	0 (0)	0 (0)	0 (0)	0 (0)
	Actinobacteria	126 (0.184)	409 (0.583)	11,113 (16.77)	344 (0.490)
	Coriobacteriia	2 (0.003)	33 (0.047)	175 (0.264)	0 (0)
	Rubrobacteria	15 (0.022)	3 (0.004)	8 (0.012)	0 (0)
	Thermoleophilia	7 (0.010)	3 (0.004)	2 (0.003)	0 (0)
Armatimonadetesa Bacteroidetes	Fimbriimonadia	0 (0)	9 (0.013)	0 (0)	0 (0)
	Bacteroidia	0 (0)	7 (0.010)	10 (0.015)	53 (0.0754)
	Chitinophagia	0 (0)	14 (0.020)	0 (0)	737 (1.049)
	Cytophagia	0 (0)	10 (0.014)	0 (0)	0 (0)
	Flavobacteriia	2 (0.003)	0 (0)	2 (0.003)	85 (0.121)
	Sphingobacteriia	4 (0.006)	0 (0)	0 (0)	4 (0.006)
Chlamydiae	Chlamydiia	0 (0)	0 (0)	0 (0)	0 (0)
Chloroflexi	Chloroflexia	0 (0)	0 (0)	0 (0)	0 (0)
	Thermomicrobia	0 (0)	0 (0)	0 (0)	0 (0)
Deinococcus-Thermus	Deinococci	0 (0)	13 (0.019)	0 (0)	10 (0.014)
Firmicutes	Bacilli	66,978 (98.01)	66,606 (94.925)	48,609 (73.34)	59,836 (85.15)
	Clostridia	602 (0.881)	1181 (1.683)	5524 (8.338)	596 (0.848)
	Erysipelotrichia	18 (0.026)	8 (0.011)	385 (0.581)	0 (0)
	Negativicutes	2 (0.003)	0	10 (0.015)	0 (0)
	Tissierellia	0 (0)	26 (0.037)	0 (0)	71 (0.101)
	Fusobacteriia	0 (0)	0 (0)	0 (0)	141 (0.201)
Gemmatimonadetes	Gemmatimonadetes	0 (0)	0 (0)	0 (0)	0 (0)
Planctomycete	Planctomycetia	0 (0)	0 (0)	0 (0)	0 (0)
Proteobacteria	Alphaproteobacteria	376 (0.550)	934 (1.331)	324 (0.489)	373 (0.531)
	Betaproteobacteria	38 (0.056)	248 (0.353)	29 (0.044)	774 (1.101)
	Deltaproteobacteria	3 (0.004)	37 (0.053)	5 (0.008)	15 (0.021)
	Gammaproteobacteria	160 (0.234)	615 (0.876)	54 (0.082)	7232 (10.292)
Verrucomicrobi	Verrucomicrobiae	0 (0)	11 (0.0157)	0 (0)	0 (0)
Unclassified	Unclassified	0 (0)	0 (0)	1 (0.002)	0 (0)
Total		68,338	70,167	66,254	70,271

**Table 2:** Abundance of bacterial 16S rDNA sequences (*n* = 212,094) identified from the jejunum microbiota of Cobb 500 broiler chickens.

Phylum	Class	Abundance of Sequence (No. of Sequence [%]) at Day:			
		Day 5	Day 15	Day 25	Day 35
Acidobacteria	Acidobacteriia	4 (0.0090)	0 (0)	3 (0.0056)	0 (0)
	Blastocatellia	0 (0)	0 (0)	0 (0)	0 (0)
Actinobacteria	Acidimicrobiia	0 (0)	5 (0.0092)	1 (0.0019)	5 (0.0082)
	Actinobacteria	672 (1.52)	497 (0.917)	2826 (5.31)	33181 (54.89)
	Coriobacteriia	24 (0.054)	14 (0.026)	2 (0.0038)	1 (0.0017)
	Rubrobacteria	105 (0.237)	20 (0.0369)	0 (0)	110 (0.182)
	Thermoleophilia	22 (0.0498)	18 (0.033)	3 (0.0056)	43 (0.071)
Armatimonadetesa	Fimbriimonadia	0 (0)	0 (0)	1 (0.0019)	0 (0)
Bacteroidetes	Bacteroidia	9 (0.02034)	32 (0.059)	6 (0.0112)	4 (0.0066)
	Chitinophagia	5 (0.0113)	0 (0)	0 (0)	12 (0.0199)
	Cytophagia	0 (0)	21 (0.039)	2 (0.0038)	0 (0)
	Flavobacteriia	15 (0.034)	17 (0.031)	8 (0.015)	1 (0.0017)
	Sphingobacteriia	0 (0)	24 (0.044)	5 (0.0094)	0 (0)
Chlamydiae	Chlamydiia	0 (0)	4 (0.0073)	0 (0)	0 (0)
Chloroflexi	Chloroflexia	4 (0.0090)	0 (0)	0 (0)	0 (0)
	Thermomicrobia	6 (0.0136)	0 (0)	0 (0)	64 (0.106)
Deinococcus-Thermus	Deinococci	0 (0)	0 (0)	1 (0.0019)	302 (0.4996)
Firmicutes	Bacilli	39,274 (88.83)	50,466 (93.09)	49,512 (93.03)	23,605 (39.05)
Firmicutes	Clostridia	2530 (5.72)	787 (1.45)	215 (0.404)	2028 (3.35)
	Erysipelotrichia	72 (0.162)	11 (0.020)	13 (0.024)	7 (0.0116)
	Negativicutes	4 (0.0090)	0 (0)	0 (0)	19 (0.0314)
	Tissierellia	27 (0.061)	20 (0.0369)	0 (0)	188 (0.311)
	Fusobacteriia	0 (0)	0 (0)	0 (0)	3 (0.00496)
Gemmatimonadetes	Gemmatimonadetes	3 (0.0068)	0 (0)	0 (0)	4 (0.0067)
Planctomycete	Planctomycetia	3 (0.0068)	0 (0)	0 (0)	0 (0)
Proteobacteria	Alphaproteobacteria	1067 (2.413)	1351 (2.49)	399 (0.75)	449 (0.743)
	Betaproteobacteria	59 (0.133)	378 (0.697)	29 (0.054)	307 (0.508)
	Deltaproteobacteria	13 (0.029)	41 (0.0756)	0 (0)	18 (0.0298)
	Gammaproteobacteria	292 (0.660)	503 (0.928)	196 (0.368)	97 (0.160)
Verrucomicrobi	Verrucomicrobiae	0 (0)	0 (0)	0 (0)	0 (0)
Unclassified	Unclassified	1 (0.0023)	3 (0.0055)	0 (0)	1(0.0017)
Total		44,211	54,212	53,222	60,449

**Table 3:** Abundance of bacterial 16S rDNA sequences (*n* = 200,624) identified from the ileum microbiota of Cobb 500 broiler chicken.

Phylum	Class	Abundance of Sequence (No. of Sequence [%]) at Day:			
		Day 5	Day 15	Day 25	Day 35
Acidobacteria	Acidobacteriia	1 (0.0017)	2 (0.0042)	6 (0.0125)	1 (0.0022)
	Blastocatellia	0 (0)	0 (0)	0 (0)	0 (0)
Actinobacteria	Acidimicrobiia	0 (0)	3 (0.0063)	10 (0.0208)	0 (0)
	Actinobacteria	306 (0.512)	730 (1.542)	5581 (11.62)	5195 (11.414)
	Coriobacteriia	1 (0.0017)	30 (0.063)	4 (0.0083)	2 (0.004)
	Rubrobacteria	7 (0.0117)	17 (0.036)	37 (0.077)	5 (0.011)
	Thermoleophilia	0 (0)	20 (0.042)	35 (0.073)	5 (0.011)
Armatimonadetesa	Fimbriimonadia	0 (0)	4 (0.0084)	0 (0)	0 (0)
Bacteroidetes	Bacteroidia	2 (0.0033)	18 (0.038)	20 (0.042)	4 (0.009)
	Chitinophagia	0 (0)	12 (0.025)	3 (0.0062)	2 (0.004)
	Cytophagia	0 (0)	11 (0.023)	0 (0)	0 (0)
	Flavobacteriia	2 (0.0033)	0 (0)	0 (0)	0 (0)
	Sphingobacteriia	0 (0)	1 (0.0021)	3 (0.0062)	0 (0)
Chlamydiae	Chlamydiia	0 (0)	1 (0.0021)	0 (0)	0 (0)
Chloroflexi	Chloroflexia	0 (0)	0 (0)	0 (0)	0 (0)
	Thermomicrobia	2 (0.0033)	8 (0.017)	5 (0.010)	1 (0.002)
Deinococcus-Thermus	Deinococci	0 (0)	0 (0)	0 (0)	35 (0.077)
Firmicutes	Bacilli	58,731 (98.327)	41,718 (88.13)	20,643 (42.97)	30,864 (67.81)
Firmicutes	Clostridia	481 (0.805)	2806 (5.927)	11,766 (24.49)	8474 (18.62)
	Erysipelotrichia	7 (0.0117)	34 (0.072)	84 (0.174)	9 (0.020)
	Negativicutes	0 (0)	3 (0.0063)	4 (0.0083)	5 (0.011)
	Tissierellia	5 (0.0084)	4 (0.0084)	49 (0.102)	2 (0.004)
	Fusobacteriia	0 (0)	0 (0)	0 (0)	0 (0)
Gemmatimonadetes	Gemmatimonadetes	0 (0)	0 (0)	0 (0)	0 (0)
Planctomycete	Planctomycetia	0 (0)	0 (0)	2 (0.0042)	0 (0)
Proteobacteria	Alphaproteobacteria	113 (0.189)	761 (1.608)	2044 (4.25)	400 (0.879)
	Betaproteobacteria	7 (0.0117)	137 (0.289)	7401 (15.41)	493 (1.083)
	Deltaproteobacteria	5 (0.0084)	7 (0.015)	10 (0.021)	2 (0.004)
	Gammaproteobacteria	58 (0.097)	1012 (2.138)	331 (0.689)	17 (0.037)
Verrucomicrobi	Verrucomicrobiae	0 (0)	0 (0)	0 (0)	0 (0)
Unclassified	Unclassified	2 (0.0033)	0 (0)	1 (0.0021)	0 (0)
Total		59,730	47,339	48,039	45,516

**Table 4:** Abundance of bacterial 16S rDNA sequences (*n* = 208,941) identified from the cecum microbiota of Cobb 500 broiler chicken.

Phylum	Class	Abundance of Sequence (No. of Sequence [%]) at Day:			
		Day 5	Day 15	Day 25	Day 35
Acidobacteria	Acidobacteriia	0 (0)	0 (0)	0 (0)	0 (0)
	Blastocatellia	0 (0)	0 (0)	0 (0)	0 (0)
Actinobacteria	Acidimicrobiia	0 (0)	0 (0)	0 (0)	0 (0)
	Actinobacteria	0 (0)	0 (0)	6 (0.014)	411 (0.818)
	Coriobacteriia	224 (0.0404)	860 (1.448)	238 (0.543)	948 (1.89)
	Rubrobacteria	0 (0)	0 (0)	0 (0)	0 (0)
	Thermoleophilia	0 (0)	0 (0)	0 (0)	0 (0)
Armatimonadetesa	Fimbriimonadia	0 (0)	0 (0)	0 (0)	0 (0)
Bacteroidetes	Bacteroidia	107 (0.193)	33 (0.056)	1 (0.0023)	71 (0.141)
	Chitinophagia	0 (0)	0 (0)	0 (0)	0 (0)
	Cytophagia	0 (0)	0 (0)	0 (0)	0 (0)
	Flavobacteriia	0 (0)	0 (0)	0 (0)	0 (0)
	Sphingobacteriia	0 (0)	0 (0)	0 (0)	0 (0)
Chlamydiae	Chlamydiia	0 (0)	0 (0)	0 (0)	0 (0)
Chloroflexi	Chloroflexia	0 (0)	0 (0)	0 (0)	0 (0)
	Thermomicrobia	0 (0)	0 (0)	0 (0)	0 (0)
Deinococcus-Thermus	Deinococci	0 (0)	0 (0)	0 (0)	0 (0)
Firmicutes	Bacilli	8839 (15.93)	3351 (5.64)	1302 (2.97)	282 (0.561)
Firmicutes	Clostridia	44,734 (80.61)	54,746 (92.19)	40,049 (91.36)	47,313 (94.19)
	Erysipelotrichia	1575 (2.838)	280 (0.472)	2233 (5.09)	1170 (2.33)
	Negativicutes	0 (0)	0 (0)	0 (0)	14 (0.028)
	Tissierellia	0 (0)	0 (0)	0 (0)	0 (0)
	Fusobacteriia	0 (0)	0 (0)	0 (0)	0 (0)
Gemmatimonadetes	Gemmatimonadetes	0 (0)	0 (0)	0 (0)	0 (0)
Planctomycete	Planctomycetia	0 (0)	0 (0)	0 (0)	0 (0)
Proteobacteria	Alphaproteobacteria	1 (0.0018)	0 (0)	1 (0.0023)	3 (0.0060)
	Betaproteobacteria	0 (0)	0 (0)	0 (0)	1 (0.0020)
	Deltaproteobacteria	0 (0)	0 (0)	0 (0)	0 (0)
	Gammaproteobacteria	12 (0.032)	0 (0)	7 (0.016)	16 (0.032)
Verrucomicrobi	Verrucomicrobiae	0 (0)	113 (0.191)	0 (0)	0 (0)
Unclassified	Unclassified	0 (0)	0 (0)	0 (0)	0 (0)
Total		55,492	59,383	43,837	50,229

**Table 5:** *P*-value distribution of 16S rDNA gene sequence libraries compared the abundance differences of microbial communities among samples from different segments for Cobb 500 Broiler chickens.

Class	*P*-Value					
	Duodenum– Jejunum	Duodenum– Ileum	Ileum– Jejunum	Caecum– Duodenum	Caecum– Ileum	Caecum– Jejunum
Acidimicrobiia	0.025	0.146	0.862		0.180	0.039
Acidobacteriia	0.772	0.275	0.684	0.163	0.027	0.082
Actinobacteria	0.683	0.957	0.536	0.300	0.034	0.258
Alphaproteobacteria	0.193	0.628	0.918	0.002	0.038	0.003
Bacilli	0.005	0.018	0.879	0.001	0.002	0.001
Bacteroidia	0.836	0.785	0.892	0.099	0.054	0.073
Betaproteobacteria	0.825	0.513	0.370	0.096	0.284	0.020
Blastocatellia	0.351	0.367		0.349		
Chitinophagia	0.345	0.363	0.954	0.304	0.084	0.168
Chlamydiia	0.351	0.493	0.591		1.000	0.356
Chloroflexia	0.351		0.377			0.356
Clostridia	0.804	0.143	0.080	0.000	0.000	0.000
Coriobacteriia	0.338	0.338	0.930	0.006	0.007	0.006
Cytophagia	0.757	0.910	0.703	0.349	0.335	0.265
Deinococci	0.537	0.841	0.482	0.059	0.335	0.334
Deltaproteobacteria	0.854	0.241	0.164	0.027	0.003	0.026
Erysipelotrichia	0.645	0.659	0.769	0.005	0.006	0.005
Fimbriimonadia	0.599	0.765	0.569	0.349	0.335	1.000
Flavobacteriia	0.769	0.306	0.014	0.290	0.505	0.006
Fusobacteriia	0.498	0.367	0.250	0.349		0.259
Gammaproteobacteria	0.491	0.535	0.777	0.228	0.110	0.004
Gemmatimonadetes	0.066		0.085			0.101
Negativicutes	0.781	0.958	0.666	0.811	0.816	0.766
Planctomycetia	0.124	0.243	1.000		0.505	0.259
Rubrobacteria	0.048	0.188	0.159	0.022	0.023	0.045
Sphingobacteriia	0.582	0.688	0.341	0.042	0.152	0.199
Thermoleophilia	0.023	0.124	0.689	0.015	0.049	0.015
Thermomicrobia	0.237	0.010	0.513		0.008	0.283
Tissierellia	0.677	0.810	0.447	0.131	0.215	0.192
Verrucomicrobiae	0.351	0.367		0.570	0.335	0.356
Unclassified	0.001	0.000	0.782	0.204	0.005	0.019

**Table 6:** *P*-value distribution of 16S rDNA gene sequence libraries compared the abundance differences of microbial communities among samples from different age periods for Cobb 500 Broiler chickens.

Class	*P*-Value					
	Day 5–15	Day 5–25	Day 5–35	Day 15–25	Day 15–35	Day 25–35
Acidimicrobiia	0.105	0.277	0.360	0.763	0.826	0.749
Acidobacteriia	0.330	0.302	0.159	0.075	1.000	0.023
Actinobacteria	0.654	0.055	0.223	0.069	0.220	0.716
Alphaproteobacteria	0.438	0.602	0.780	0.955	0.121	0.637
Bacilli	0.835	0.536	0.582	0.699	0.748	0.932
Bacteroidia	0.766	0.522	0.907	0.099	0.729	0.171
Betaproteobacteria	0.054	0.432	0.020	0.473	0.216	0.645
Blastocatellia	0.333	0.339	0.360			
Chitinophagia	0.209	0.813	0.340	0.140	0.415	0.344
Chlamydiia	0.191			0.201	0.141	
Chloroflexia	0.333	0.339	0.360			
Clostridia	0.887	0.910	0.854	0.956	0.938	0.957
Coriobacteriia	0.578	0.664	0.681	0.685	0.919	0.732
Cytophagia	0.023	0.241		0.024	0.021	0.249
Deinococci	0.333	0.491	0.235	0.431	0.257	0.201
Deltaproteobacteria	0.152	0.765	0.693	0.124	0.318	0.490
Erysipelotrichia	0.521	0.699	0.815	0.330	0.680	0.718
Fimbriimonadia	0.125	0.491		0.174	0.095	0.499
Flavobacteriia	0.888	0.637	0.636	0.738	0.615	0.583
Fusobacteria			0.317		0.335	0.321
Gammaproteobacteria	0.068	0.865	0.531	0.101	0.682	0.510
Gemmatimonadetes	0.119	0.250	0.783		0.373	0.370
Negativicutes	0.658	0.509	0.042	0.267	0.035	0.172
Planctomycetia	0.119	1.000	0.250	0.234		0.249
Rubrobacteria	0.516	0.547	0.929	0.865	0.712	0.707
Sphingobacteriia	0.507	0.576	0.940	0.613	0.550	0.692
Thermoleophilia	0.754	0.782	0.746	0.950	0.860	0.903
Thermomicrobia	0.956	0.750	0.557	0.787	0.518	0.511
Tissierellia	0.714	0.733	0.191	0.937	0.225	0.243
Verrucomicrobiae	0.287			0.288	0.277	
Unclassified	0.875	0.701	0.870	0.843	0.844	0.712

**Table 7:** Abundance of bacterial 16S rDNA sequences (*n* = 391,488) isolated from the duodenum microbiota of local Omani chicken.

Phylum	Class	Abundance of Sequence (No. of Sequence [%]) at Day:			
		Day 5	Day 15	Day 25	Day 35
Acidobacteria	Acidobacteriia	0 (0)	6 (0.007)	1 (0.001)	0 (0)
	Blastocatellia	0 (0)	0 (0)	3 (0.003)	0 (0)
Actinobacteria	Acidimicrobiia	4 (0.004)	0 (0)	1 (0.001)	0 (0)
	Actinobacteria	363 (0.400)	506 (0.548)	550 (0.497)	2360 (2.42)
	Coriobacteriia	5 (0.006)	4 (0.004)	33 (0.030)	1 (0.001)
	Rubrobacteria	5 (0.006)	3 (0.003)	6 (0.005)	8 (0.008)
	Thermoleophilia	1 (0.001)	0 (0)	8 (0.007)	1 (0.001)
Armatimonadetes	Fimbriimonadia	0 (0)	0 (0)	5 (0.005)	0 (0)
Bacteroidetes	Bacteroidia	115 (0.127)	587 (0.636)	334 (0.302)	65 (0.067)
	Chitinophagia	81 (0.089)	30 (0.032)	107 (0.097)	25 (0.026)
	Cytophagia	10 (0.011)	0 (0)	17 (0.015)	22 (0.022)
	Flavobacteriia	46 (0.051)	11 (0.011)	404 (0.365)	74 (0.076)
	Sphingobacteriia	0 (0)	6 (0.007)	23 (0.020)	0 (0)
Chlamydiae	Chlamydiia	0 (0)	0 (0)	3 (0.003)	0 (0)
Chloroflexi	Anaerolineae	11 (0.012)	0 (0)	0 (0)	0 (0)
	Ktedonobacteria	0 (0)	2 (0.002)	0 (0)	0 (0)
	Thermomicrobia	9 (0.010)	0 (0)	1 (0.001)	3 (0.003)
Deinococcus-Thermus	Deinococci	16 (0.018)	61 (0.066)	27 (0.024)	38 (0.039)
Firmicutes	Bacilli	62,254 (68.52)	80,193 (86.91)	67,760 (61.19)	90,628 (92.83)
	Clostridia	23,473 (25.84)	6131 (6.64)	29,626 (26.75)	871 (0.892)
	Erysipelotrichia	511 (0.562)	87 (0.094)	171 (0.154)	15 (0.015)
	Negativicutes	13 (0.014)	40 (0.043)	15 (0.014)	66 (0.068)
	Tissierellia	5 (0.006)	3 (0.003)	69 (0.062)	48 (0.049)
Fusobacteria	Fusobacteriia	0 (0)	6 (0.007)	43 (0.039)	11 (0.011)
Gemmatimonadetes	Gemmatimonadetes	0 (0)	0 (0)	40 (0.036)	0 (0)
Planctomycetes	Planctomycetia	3 (0.003)	0 (0)	15 (0.016)	0 (0)
Proteobacteria	Alphaproteobacteria	1503 (1.65)	1677 (1.82)	1576 (1.42)	1986 (2.03)
	Betaproteobacteria	799 (0.879)	818 (0.887)	1576 (1.42)	746 (0.764)
	Deltaproteobacteria	32 (0.053)	76 (0.082)	43 (0.039)	66 (0.068)
	Epsilonproteobacteria	0 (0)	1 (0.001)	6 (0.005)	0 (0)
	Gammaproteobacteria	1551 (1.71)	1970 (2.14)	8237 (7.44)	550 (0.563)
Spirochaetes	Spirochaetia	0 (0)	0 (0)	3 (0.003)	0 (0)
Tenericutes	Mollicutes	0 (0)	0 (0)	21 (0.019)	0 (0)
Verrucomicrobia	Opitutae	0 (0)	0 (0)	2 (0.002)	0 (0)
	Verrucomicrobiae	10 (0.011)	15 (0.016)	0 (0)	0 (0)
Unclassified	Unclassified	33 (0.036)	34 (0.037)	14 (0.013)	47 (0.048)
Total		90,853	92,267	11,0737	97,631

**Table 8:** Abundance of bacterial 16S rDNA sequences (*n* = 307,792) isolated from the jejunum microbiota of Omani chicken.

Phylum	Class	Abundance of Sequence (No. of Sequence [%]) at Day:			
		Day 5	Day 15	Day 25	Day 35
Acidobacteria	Acidobacteriia	0 (0)	0 (0)	0 (0)	0 (0)
	Blastocatellia	0 (0)	0 (0)	0 (0)	0 (0)
Actinobacteria	Acidimicrobiia	1 (0.001)	0 (0)	0 (0)	0 (0)
	Actinobacteria	3971 (5.25)	412 (0.612)	750 (0.890)	4369 (5.42)
	Coriobacteriia	14 (0.019)	5 (0.007)	21 (0.025)	13 (0.016)
	Rubrobacteria	12 (0.016)	9 (0.013)	7 (0.008)	18 (0.022)
	Thermoleophilia	3 (0.004)	10 (0.015)	3 (0.004)	8 (0.01)
Armatimonadetes	Fimbriimonadia	0 (0)	0 (0)	0 (0)	0 (0)
Bacteroidetes	Bacteroidia	5 (0.007)	212 (0.315)	128 (0.152)	77 (0.096)
	Chitinophagia	1 (0.001)	1 (0.001)	0 (0)	0 (0)
	Cytophagia	0 (0)	0 (0)	0 (0)	0 (0)
	Flavobacteriia	2 (0.003)	1 (0.001)	0 (0)	1 (0.001)
	Sphingobacteriia	3 (0.004)	0 (0)	0 (0)	0 (0)
Chlamydiae	Chlamydiia	0 (0)	0 (0)	0 (0)	0 (0)
Chloroflexi	Anaerolineae	0 (0)	0 (0)	0 (0)	0 (0)
	Ktedonobacteria	0 (0)	0 (0)	0 (0)	0 (0)
	Thermomicrobia	22 (0.029)	3 (0.004)	1 (0.001)	2 (0.002)
Deinococcus-Thermus	Deinococci	4 (0.005)	0 (0)	2 (0.002)	14 (0.017)
Firmicutes	Bacilli	67,339 (89.06)	60,051 (89.16)	80,018 (94.92)	69,329 (86.09)
	Clostridia	3393 (4.49)	5796 (8.61)	3081 (3.65)	6210 (7.71)
	Erysipelotrichia	81 (0.107)	98 (0.146)	33 (0.039)	50 (0.062)
	Negativicutes	5 (0.006)	4 (0.006)	9 (0.011)	83 (0.103)
	Tissierellia	8 (0.010)	3 (0.004)	0 (0)	1 (0.001)
Fusobacteria	Fusobacteriia	0 (0)	3 (0.004)	0 (0)	0 (0)
Gemmatimonadetes	Gemmatimonadetes	0 (0)	0 (0)	0 (0)	0 (0)
Planctomycetes	Planctomycetia	0 (0)	0 (0)	0 (0)	0 (0)
Proteobacteria	Alphaproteobacteria	300 (0.397)	247 (0.368)	112 (0.133)	91 (0.113)
	Betaproteobacteria	48 (0.064)	83 (0.123)	29 (0.034)	65 (0.081)
	Deltaproteobacteria	0 (0)	12 (0.018)	3 (0.004)	4 (0.005)
	Epsilonproteobacteria	0 (0)	0 (0)	0 (0)	0 (0)
	Gammaproteobacteria	399 (0.528)	398 (0.591)	98 (0.116)	123 (0.153)
Spirochaetes	Spirochaetia	0 (0)	0 (0)	0 (0)	0 (0)
Tenericutes	Mollicutes	0 (0)	0 (0)	0 (0)	0 (0)
Verrucomicrobia	Opitutae	0 (0)	0 (0)	0 (0)	0 (0)
	Verrucomicrobiae	0 (0)	0 (0)	1 (0.001)	74 (0.092)
Unclassified	Unclassified	2 (0.003)	1 (0.001)	1 (0.001)	1 (0.001)
Total		75,613	67,349	84,297	80,533

**Table 9:** Abundance of bacterial 16S rDNA sequences (*n* = 268,837) isolated from the ileum microbiota of Omani chicken.

Phylum	Class	Abundance of Sequence (No. of Sequence [%]) at Day:			
		Day 5	Day 15	Day 25	Day 35
Acidobacteria	Acidobacteriia	0 (0)	0 (0)	0 (0)	0 (0)
	Blastocatellia	0 (0)	0 (0)	0 (0)	0 (0)
Actinobacteria	Acidimicrobiia	2 (0.003)	0 (0)	0 (0)	0 (0)
	Actinobacteria	948 (1.39)	692 (0.831)	509 (0.759)	1754 (3.48)
	Coriobacteriia	17 (0.025)	1 (0.001)	21 (0.031)	12 (0.024)
	Rubrobacteria	11 (0.016)	2 (0.002)	15 (0.022)	5 (0.01)
	Thermoleophilia	12 (0.018)	1 (0.001)	10 (0.015)	2 (0.004)
Armatimonadetes	Fimbriimonadia	0 (0)	0 (0)	1 (0.001)	0 (0)
Bacteroidetes	Bacteroidia	17 (0.025)	23 (0.028)	231 (0.345)	11 (0.022)
	Chitinophagia	12 (0.018)	0 (0)	6 (0.009)	1 (0.002)
	Cytophagia	8 (0.012)	0 (0)	0 (0)	0 (0)
	Flavobacteriia	2 (0.003)	4	4 (0.006)	3 (0.006)
	Sphingobacteriia	7 (0.010)	0 (0)	2 (0.003)	1 (0.002)
Chlamydiae	Chlamydiia	0 (0)	0 (0)	0 (0)	0 (0)
Chloroflexi	Anaerolineae	0 (0)	0 (0)	0 (0)	0 (0)
	Ktedonobacteria	0 (0)	0 (0)	0 (0)	0 (0)
	Thermomicrobia	13 (0.019)	8 (0.010)	5 (0.007)	1 (0.002)
Deinococcus-Thermus	Deinococci	1 (0.001)	0 (0)	4 (0.006)	1 (0.002)
Firmicutes	Bacilli	58,866 (86.44)	70,958 (85.19)	47,702 (71.15)	43,183 (85.68)
	Clostridia	6286 (9.23)	10,840 (13.01)	17,497 (26.10)	5187 (10.29)
	Erysipelotrichia	143 (0.210)	56 (0.067)	93 (0.139)	26 (0.052)
	Negativicutes	11 (0.016)	1 (0.001)	15 (0.022)	12 (0.024)
	Tissierellia	17 (0.025)	2 (0.002)	3 (0.004)	0 (0)
Fusobacteria	Fusobacteriia	0 (0)	1 (0.001)	5 (0.007)	0 (0)
Gemmatimonadetes	Gemmatimonadetes	0 (0)	0	0 (0)	0 (0)
Planctomycetes	Planctomycetia	0 (0)	0	1 (0.001)	0 (0)
Proteobacteria	Alphaproteobacteria	500 (0.734)	217 (0.261)	432 (0.644)	110 (0.218)
	Betaproteobacteria	180 (0.264)	34 (0.041)	120 (0.179)	33 (0.065)
	Deltaproteobacteria	14 (0.020)	1 (0.001)	11 (0.016)	2 (0.004)
	Epsilonproteobacteria	0 (0)	0 (0)	0 (0)	0 (0)
	Gammaproteobacteria	1032 (1.52)	449 (0.539)	355 (0.529)	53 (0.105)
Spirochaetes	Spirochaetia	0 (0)	0 (0)	0 (0)	0 (0)
Tenericutes	Mollicutes	0 (0)	0 (0)	0 (0)	0 (0)
Verrucomicrobia	Opitutae	0 (0)	0 (0)	0 (0)	0 (0)
	Verrucomicrobiae	0 (0)	0 (0)	1 (0.001)	1 (0.002)
Unclassified	Unclassified	2 (0.003)	0 (0)	3 (0.004)	2 (0.004)
Total		68,101	83,290	67,046	50,400

**Table 10:** Abundance of bacterial 16S rDNA sequences (*n* = 266,937) isolated from the cecum microbiota of Omani chicken.

Phylum	Class	Abundance of Sequence (No. of Sequence [%]) at Day:			
		Day 5	Day 15	Day 25	Day 35
Acidobacteria	Acidobacteriia	0 (0)	0 (0)	0 (0)	0 (0)
	Blastocatellia	0 (0)	0 (0)	0 (0)	0 (0)
Actinobacteria	Acidimicrobiia	0 (0)	0 (0)	0 (0)	0 (0)
	Actinobacteria	13 (0.017)	73 (0.125)	1718 (2.75)	142 (0.203)
	Coriobacteriia	59 (0.077)	55 (0.094)	151 (0.242)	270 (0.386)
	Rubrobacteria	0 (0)	0 (0)	0 (0)	0 (0)
	Thermoleophilia	0 (0)	0 (0)	0 (0)	0 (0)
Armatimonadetes	Fimbriimonadia	0 (0)	0 (0)	0 (0)	0 (0)
Bacteroidetes	Bacteroidia	0 (0)	916 (1.57)	3573 (5.72)	8685 (12.42)
	Chitinophagia	0 (0)	0 (0)	0 (0)	1 (0.001)
	Cytophagia	0 (0)	0 (0)	0 (0)	0 (0)
	Flavobacteriia	0 (0)	0 (0)	0 (0)	1 (0.001)
	Sphingobacteriia	0 (0)	0 (0)	0 (0)	0 (0)
Chlamydiae	Chlamydiia	0 (0)	0 (0)	0 (0)	0 (0)
Chloroflexi	Anaerolineae	0 (0)	0 (0)	0 (0)	0 (0)
	Ktedonobacteria	0 (0)	0 (0)	0 (0)	0 (0)
	Thermomicrobia	0 (0)	0 (0)	0 (0)	0 (0)
Deinococcus-Thermus	Deinococci	0 (0)	0 (0)	0 (0)	0 (0)
Firmicutes	Bacilli	11,773 (15.45)	306 (0.525)	410 (0.656)	518 (0.741)
	Clostridia	60,256 (79.08)	54,535 (93.48)	53,433 (85.55)	48,355 (69.14)
	Erysipelotrichia	1534 (2.01)	1753 (3.00)	1508 (2.42)	780 (1.11)
	Negativicutes	0 (0)	1 (0.002)	9 (0.014)	7102 (10.15)
	Tissierellia	0 (0)	0 (0)	0 (0)	0 (0)
Fusobacteria	Fusobacteriia	0 (0)	6	0 (0)	0 (0)
Gemmatimonadetes	Gemmatimonadetes	0 (0)	0 (0)	0 (0)	0 (0)
Planctomycetes	Planctomycetia	0 (0)	0 (0)	0 (0)	0 (0)
Proteobacteria	Alphaproteobacteria	0 (0)	1 (0.002)	4 (0.006)	25 (0.036)
	Betaproteobacteria	1 (0.001)	3 (0.005)	4 (0.006)	159 (0.227)
	Deltaproteobacteria	0 (0)	1 (0.002)	0 (0)	107 (0.153)
	Epsilonproteobacteria	0 (0)	0 (0)	0 (0)	0 (0)
	Gammaproteobacteria	2564 (3.36)	687 (1.18)	1649 (2.64)	2630 (3.76)
Spirochaetes	Spirochaetia	0 (0)	0 (0)	0 (0)	0 (0)
Tenericutes	Mollicutes	0 (0)	0 (0)	0 (0)	0 (0)
Verrucomicrobia	Opitutae	0 (0)	0 (0)	0 (0)	0 (0)
	Verrucomicrobiae	0 (0)	0 (0)	0 (0)	1166 (1.67)
Unclassified	Unclassified	0 (0)	0 (0)	0 (0)	0 (0)
Total		76,200	58,337	62,459	69,941

**Table 11:** *P*-value distribution of 16S rDNA gene sequence libraries compared the abundance differences of microbial communities among samples from different segments for local Omani chicken.

Class	*P*-Value					
	Duodenum– Jejunum	Duodenum– Ileum	Ileum– Jejunum	Caecum– Duodenum	Caecum– Ileum	Caecum– Jejunum
Acidimicrobiia	0.497	0.654	1	0.212	1	0.509
Acidobacteriia	0.238	0.258		0.249		
Actinobacteria	0.148	0.755	0.239	0.849	0.132	0.629
Alphaproteobacteria	0	0	0.309	0	0.009	0.008
Anaerolineae	0.347	0.345		0.359		
Bacilli	0.629	0.166	0.014	0.001	0	0.001
Bacteroidia	0.306	0.140	0.554	0.099	0.054	0.073
Betaproteobacteria	0.001	0.001	0.501	0.002	0.887	0.591
Blastocatellia	0.255	0.253		0.278		
Chitinophagia	0.004	0.010	0.138	0.006	1	0.122
Chlamydiia	0.255	0.253		0.278		
Clostridia	0.149	0.684	0.071	0.002	0.001	0
Coriobacteriia	0.608	0.666	0.895	0.01	0.022	0.016
Cytophagia	0.017	0.067	0.348	0.012		0369
Deinococci	0.010	0.007	0.278	0.005	0.113	0.061
Deltaproteobacteria	0.003	0.003	0.728	0.781	0.499	0.540
Epsilonproteobacteria	0.197	0.223		0.225		
Erysipelotrichia	0.343	0.462	0.819	0.004	0.005	0.004
Fimbriimonadia	0.347	0.567	1	0.359		1
Flavobacteriia	0.111	0.134	0.007	0.110	0.167	0.002
Fusobacteriia	0.115	0.146	0.753	0.190	0.633	0.747
Gammaproteobacteria	0.079	0.119	0.503	0.904	0.008	0.007
Gemmatimonadetes	0.347	0.345		0.359		
Ktedonobacteria	0.503	0.502		0.519		
Mollicutes	0.347	0.345		0.359		
Negativicutes	0.863	0.092	0.519	0.507	0.416	0.442
Opitutae	0.503	0.502		0.519		
Planctomycetia	0.169	0.242	1	0.168		1
Rubrobacteria	0.009	0.251	0.292	0.003	0.004	0.011
Sphingobacteriia	0.225	0.586	0.398	0.140	0.259	0.102
Spirochaetia	0.255	0.253		0.278		
Thermoleophilia	0.075	0.173	0.917	0.134	0.012	0.030
Thermomicrobia	0.592	0.293	0.874	0.111	0.181	0.014
Tissierellia	0.078	0.135	0.645	0.037	0.095	0.170
Verrucomicrobiae	0.657	0.130	0.402	0.512	0.451	0.422
Unclassified	0.003	0.002	0.010	0.030	0.007	0.003

**Table 12:** *P*-value distribution of 16S rDNA gene sequence libraries compared the abundance differences of microbial communities among samples from different age periods for local Omani chickens.

Class	*P*-Value					
	Day 5–15	Day 5–25	Day 5–35	Day 15–25	Day 15–35	Day 25–35
Acidimicrobiia	0.028	0.064	0.034	1		1
Acidobacteriia	0.362	1	0.650	0.574	0.361	1
Actinobacteria	0.439	0.782		0.230	0.041	0.254
Alphaproteobacteria	0.906	0.797	0.894	0.905	0.952	0.919
Anaerolineae	0.362	0.351	0.365			
Bacilli	0.916	0.788	0.958	0.846	0.914	0.825
Bacteroidia	0.081	0.287	0.346	0.713	0.580	0.745
Betaproteobacteria	0.914	0.818	0.946	0.854	0.926	0.806
Blastocatellia		0.251		0.252		0.252
Chitinophagia	0.511	0.958	0.552	0.707	0.873	0.604
Chlamydiia		0.251		0.252		0.252
Clostridia	0.947	0.876	0.747	0.932	0.764	0.712
Coriobacteriia	0.870	0.536	0.581	0.546	0.583	0.866
Cytophagia	0.084	0.824	0.909	0.367	0.361	0.831
Deinococci	0.579	0.821	0.561	0.800	0.880	0.667
Deltaproteobacteria	0.589	0.944	0.232	0.779	0.593	0.276
Epsilonproteobacteria	0.488	0.351		0.624	0.495	0.359
Erysipelotrichia	0.863	0.936	0.583	0.911	0.665	0.675
Fimbriimonadia		0.199		0.170		0.199
Flavobacteriia	0.518	0.561	0.757	0.487	0.547	0.600
Fusobacteriia	0.005	0.250	0.365	0.758	0.595	0.552
Gammaproteobacteria	0.470	0.715	0.686	0.547	0.934	0.590
Gemmatimonadetes		0.351		0.367		
Ktedonobacteria	0.238			0.223	0.245	0.359
Mollicutes		0.351		0.367		0.359
Negativicutes	0.682	0.142	0.326	0.950	0.339	0.342
Opitutae		0.501		0.502		0.501
Planctomycetia	0.250	0.574	0.250	0.286		0.292
Rubrobacteria	0.453	0.857	0.882	0.703	0.516	0.804
Sphingobacteriia	0.618	0.717	0.183	0.653	0.599	0.325
Spirochaetia		0.251		0.252		0.252
Thermoleophilia	0.774	0.886	0.724	0.827	0.903	0.647
Thermomicrobia	0.130	0.047	0.048	0.765	0.660	0.937
Tissierellia	0.174	0.743	0.766	0.541	0.568	0.890
Verrucomicrobiae	0.746	0.593	0.294	0.564	0.314	0.313
Unclassified	0.730	0.877	0.782	0.833	0.720	0.944
